# IKK is a therapeutic target in KRAS-Induced lung cancer with disrupted p53 activity

**DOI:** 10.18632/genesandcancer.5

**Published:** 2014-01

**Authors:** Daniela S. Bassères, Aaron Ebbs, Patricia C. Cogswell, Albert S. Baldwin

**Affiliations:** ^1^ Department of Biochemistry, Chemistry Institute, University of São Paulo, São Paulo, SP, Brazil;; ^2^ Lineberger Comprehensive Cancer Center, University of North Carolina, Chapel Hill, NC;; ^3^ Department of Biology, University of North Carolina, Chapel Hill, NC.

**Keywords:** Lung cancer, KRAS, NF-κB, IKK, p53

## Abstract

Activating mutations in *KRAS* are prevalent in cancer, but therapies targeted to oncogenic RAS have been ineffective to date. These results argue that targeting downstream effectors of RAS will be an alternative route for blocking RAS-driven oncogenic pathways. We and others have shown that oncogenic RAS activates the NF-κB transcription factor pathway and that KRAS-induced lung tumorigenesis is suppressed by expression of a degradation-resistant form of the IκBα inhibitor or by genetic deletion of IKKβ or the RELA/p65 subunit of NF-κB. Here, genetic and pharmacological approaches were utilized to inactivate IKK in human primary lung epithelial cells transformed by KRAS, as well as *KRAS* mutant lung cancer cell lines. Administration of the highly specific IKKβ inhibitor Compound A (CmpdA) led to NF-κB inhibition in different *KRAS* mutant lung cells and siRNA-mediated knockdown of IKKα or IKKβ reduced activity of the NF-κB canonical pathway. Next, we determined that both IKKα and IKKβ contribute to oncogenic properties of *KRAS* mutant lung cells, particularly when p53 activity is disrupted. Based on these results, CmpdA was tested for potential therapeutic intervention in the Kras-induced lung cancer mouse model (*LSL-Kras*^G12D^) combined with loss of p53 (*LSL-Kras*^G12D^/*p53*^fl/fl^). CmpdA treatment was well tolerated and mice treated with this IKKβ inhibitor presented smaller and lower grade tumors than mice treated with placebo. Additionally, IKKβ inhibition reduced inflammation and angiogenesis. These results support the concept of targeting IKK as a therapeutic approach for oncogenic RAS-driven tumors with altered p53 activity.

## INTRODUCTION

Lung cancer is the leading cause of cancer-related deaths worldwide [1] and even though novel targeted therapies have been developed that show efficacy for a subset of patients [2,3], for the great majority of lung cancer patients effective targeted therapies are still lacking. This is the case for the 20-50% of lung cancer patients that harbor activating point mutations in the *KRAS* GTPase gene [4-6]. Therefore, identification of druggable targets in the KRAS signaling pathway could lead to novel therapeutic alternatives for lung cancer, as well as other RAS-driven cancers.

Constitutive, signal-independent activation of KRAS via mutation is not only associated with poor prognosis and therapy resistance in a variety of cancers, but is sufficient to trigger malignant transformation and drive the oncogenic phenotype [7,8]. Therefore, KRAS is a rational target for cancer therapy. Unfortunately, due to the difficulty in effectively inhibiting the biological activity of RAS proteins, approaches to directly target these proteins for therapy have been so far unsuccessful [9]. In this regard, intense efforts are being made to target known downstream effectors of RAS [10,11,12]. So far this approach has yielded limited therapeutic success, thus reflecting the need to better understand the molecular pathways triggered by oncogenic RAS.

A mechanism that is known to be important for RAS-induced oncogenesis is the activation of the transcription factor NF-κB. NF-κB is a ubiquitously expressed transcription factor that is maintained in an inactive form through interactions with the inhibitor of κB (IκB) proteins. Canonical NF-κB activation downstream of inflammatory cytokines and other inducing molecules is mediated by the IκB kinase (IKK) complex, which is comprised of a regulatory subunit (NEMO) and two catalytic subunits (IKKα and IKKβ). Once activated, the IKK complex phosphorylates IκB, which leads to its rapid ubiquitination and proteasome-mediated degradation. In this pathway, the p50-p65/RELA heterdodimer is then released and accumulates in the nucleus to regulate target gene transcription [13-17]. In the non-canonical NF-κB pathway, NIK activates an IKKα homodimer to lead to nuclear accumulation of a p52-RELB heterodimer [13,14,16,17]. Additionally, IKKβ and TBK1, IKK-related kinases can activate p65- and c-REL-containing complexes [18,19].

We previously demonstrated that NF-κB is activated downstream of oncogenic RAS and that inhibition of NF-κB leads to RAS-induced cell death [20,21]. Inhibition of NF-κB by expression of the super-repressor form of IκBα [22] or deletion of the RELA/p65 subunit of NF-κB [23] blocks KRAS-induced lung tumors. In that latter work, we demonstrated that KRAS activates the transcription factor NF-κB in lung tumors *in situ* and that loss of p65 in the tumors leads to the induction of apoptosis [23]. Barbie *et al* [24] have shown that the IKK-related kinase TBK1 is important as a survival factor in KRAS-driven cancer cells, potentially through a mechanism that involves c-REL. Duran *et al* [25] demonstrated that oncogenic KRAS can activate IKK through the signaling adaptor p62 and other studies have shown that genetic deletion of IKKβ in different cancer models suppresses RAS-induced tumorigenesis [26-28].

Here we show that pharmacological inhibition of IKKβ in primary human lung epithelial cells transformed by KRAS, as well as *KRAS* mutant lung cancer cell lines, inhibits NF-κB activity and reduces cell growth. Further analysis indicated that this response was at the level of cellular proliferation and not induction of cell death. Genetic targeting of KRAS, IKKβ or IKKα by siRNA had similar effects on NF-κB activity, reducing canonical NF-κB activation. In addition, cell growth and proliferation were also similarly affected. Nonetheless, even though NF-κB activity was reduced in all cells examined, reduced cell growth was restricted to cells with lost or disrupted p53 function. Therefore, we treated a KRAS-induced lung cancer mouse model combined with loss of the tumor suppressor p53 with a highly specific IKKβ inhibitor (Compound A, Bayer [29]). The inhibitor is well tolerated and lowers tumor burden and tumor grade. Consistent with the cell-based studies, Compound A (CmpdA) treatment reduces tumor proliferation. CmpdA also affects the tumor microenvironment, reducing the tumor-associated macrophage footprint along with reduced intratumoral vasculature. These results show that IKKα or IKKβ inhibition reduces lung cancer cell proliferation *in vitro* and pharmacological IKKβ targeting reduces lung cancer growth *in vivo*, supporting the hypothesis that IKK inhibition therapy will have clinical benefits in lung cancer as well as other cancers, particularly for patients with KRAS mutations and altered p53 activity.

## RESULTS

### IKK targeting decreases canonical NF-κB activity in KRAS mutant cell lines

**Fig 1:**
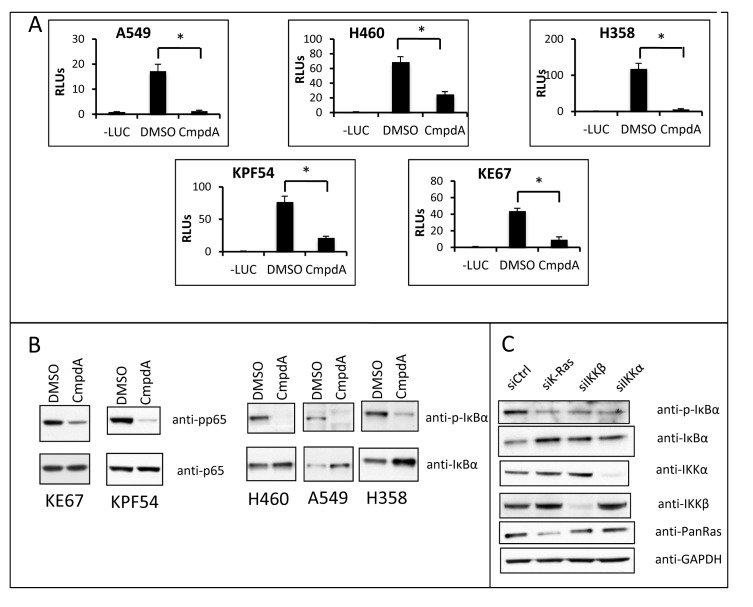
Targeting IKKβ reduces NF-κB activity in KRAS positive lung cells A) The indicated cell lines were transfected with 100ηg of an NF-κB-responsive firefly luciferase reporter vector (3x-κB-Luc) and 5ηg of Renilla luciferase vector (pRL-TK) and either treated with 0.1% DMSO or 5μM CmpdA for 16h.-LUC) negative control (cells transfected with pcDNA3 instead of 3x-κB-Luc and pRL-TK); RLUs) Relative luciferase units. B) The indicated cell lines were treated with 0.1% DMSO or 5μM CmpdA for 30 min and protein lysates were analyzed for NF-κB activity by Western Blotting. Antibodies used are indicated. C) H358 cells were transfected with the indicated siRNAs as described in methods and analyzed for NF-κB activity at 72h by Western Blotting. Antibodies used are indicated. Statistical significance was measured when appropriate by Student's *t*-test (*p<0.05) when compared to experimental control samples (DMSO). Error bars represent average ± 1s.d.

We had previously shown that oncogenic KRAS expression in low-passage primary immortalized human small airway cells correlates with increased IκB phosphorylation and NF-κB DNA binding [23]. In addition, IKK inhibition with CmpdA reduced NF-κB DNA binding activity in these genetically defined cells [23]. To further address the contribution of IKK to oncogenic RAS-driven NF-κB activity, human as well as mouse lung cancer cell lines harboring oncogenic KRAS were transfected with an NF-κB-dependent luciferase reporter and treated with CmpdA. In each experiment, the IKK inhibitor reduced NF-κB reporter activity (Fig. 1A). CmpdA also blocked phosphorylation of IKKβ substrates IκBα or p65 in these cell lines (Fig. 1B). Furthermore, when KRAS or IKKβ expression was reduced by transfecting human lung cancer cell lines with siRNA targeting either KRAS or IKKβ, NF-κB luciferase reporter activity was also inhibited, indicating that, in these cells, both KRAS and IKKβ are promoting NF-κB activity (Supplementary Fig. S1).

Based on the results showing IKKβ is involved in KRAS-induced NF-κB activation, and based on published evidence that NF-κB activation by KRAS in lung cells involves the IKK complex [23,25], we hypothesized that in human lung cells KRAS utilizes the canonical pathway to activate NF-κB. In this regard, we questioned whether siRNA-mediated inhibition of IKKα would also affect NF-κB activity in *KRAS* mutant lung cells. IKKα is typically considered to be less important in canonical NF-κB signaling as compared to IKKβ [30]. Interestingly, knockdown of IKKα in the lung cancer cells studied not only reduced NF-κB activity (Supplementary Fig. S1), but more importantly, inhibition of KRAS, IKKβ or IKKα by siRNA in H358 cells inhibited IκBα phosphorylation and degradation, a hallmark of the canonical NF-κB activation pathway (Fig. 1C).

### IKK targeting reduces proliferation of KRAS positive cells dependent on the loss of p53 function

**Fig 2:**
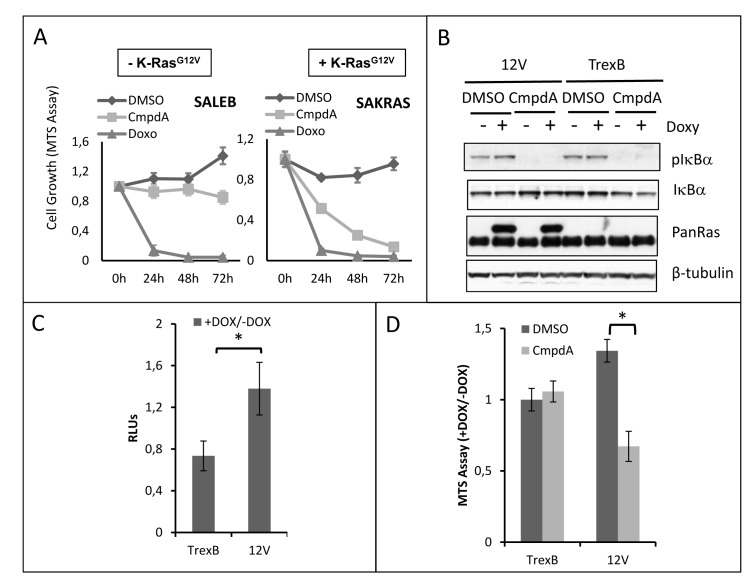
CmpdA reduces growth of lung cells in a KRAS dependent manner **A)** Primary immortalized human airway cells (SALEB) and their KRAS-transformed counterpart (SAKRAS) were treated with 0.1% DMSO or 5μM CmpdA for the indicated timepoints. 2μg/mL Doxorubicin (Doxo) was used as a positive control. Cell growth was measured using a colorimetric MTS tetrazolium assay (CellTiter 96® AQ_ueous_ One Solution Cell Proliferation Assay from Promega, Madison, WI). **B)** H1703-TrexB and H1703-G12V lung cancer cells were treated with 2μg/mL doxycycline (Doxy) for 24h where indicated to induce KRAS expression. Subsequently cells were treated with 0.1% DMSO or 5μM CmpdA for 30 min and analyzed for NF-κB activity by Western Blotting. Antibodies used are indicated. **C)** H1703-TrexB and H1703-G12V cells were transfected with 100ηg of an NF-κB-responsive firefly luciferase reporter vector (3x-κB-Luc) and 5ηg of Renilla luciferase vector (pRL-TK). Cells were induced with 2μg/mL doxycycline (+DOX) or left untreated (-DOX) for 24h and subsequently treated for 16h with either 0.1% DMSO or 5μM CmpdA as indicated. Results are expressed as the luciferase activity ratio of induced/uninduced cells (+DOX/-DOX). RLUs) Relative luciferase units. **D)** H1703-TrexB and H1703-G12V cells were treated with 2μg/mL Doxycycline (+DOX) for 24h to induce KRAS expression. Control cells were left untreated (-DOX). Subsequently, they were treated either with 0.1% DMSO or 5μM CmpdA and cell growth was measured 48h later using a colorimetric MTS tetrazolium assay (CellTiter 96® AQ_ueous_ One Solution Cell Proliferation Assay from Promega, Madison, WI). Results are expressed as growth ratio of induced/uninduced cells (+DOX/-DOX). Statistical significance in all cases was measured by Student's *t*-test (*p<0.05) when compared to experimental control samples (DMSO). Error bars represent average ± 1s.d.

Next, we examined the effects of CmpdA treatment on growth of several KRAS positive cells. Interestingly, in spite of reducing NF-κB activity in all cell lines studied, CmpdA did not affect cell growth uniformly. SAKRAS cells are very sensitive to CmpdA treatment, whereas their isogenic cells lacking oncogenic KRAS (SALEB) are less sensitive (Fig. 2A), indicating that the effect of CmpdA on cell growth is dependent on KRAS status. In order to further assess this dependency, we used H1703 lung adenocarcinoma cells, which harbor wild-type KRAS, to generate HA-tagged KRAS^G12V^-inducible H1703 human lung cancer cells. We observed that induction of KRAS^G12V^ expression with doxycycline in H1703 cells leads to increased IκBα phosphorylation and to increased NF-κB reporter activity (Figs. 2B and 2C). These effects on NF-κB activity were not observed in doxycycline-treated empty vector control cells (H1703-TrexB cells), which do not express KRAS^G12V^ (Figs. 2B and 2C). CmpdA treatment blocked IκBα phosphorylation in both KRAS^G12V^ and TrexB H1703 cells (Fig. 2B). We then used this cell line model to evaluate CmpdA sensitivity. Induction of KRAS^G12V^ expression with doxycycline in H1703 cells leads to enhanced cell growth, which is blocked by treatment with CmpdA (Fig. 2D). CmpdA had no effect on growth of H1703 TrexB cells, which do not express KRAS^G12V^ upon doxycycline administration (Fig. 2D).

**Fig 3:**
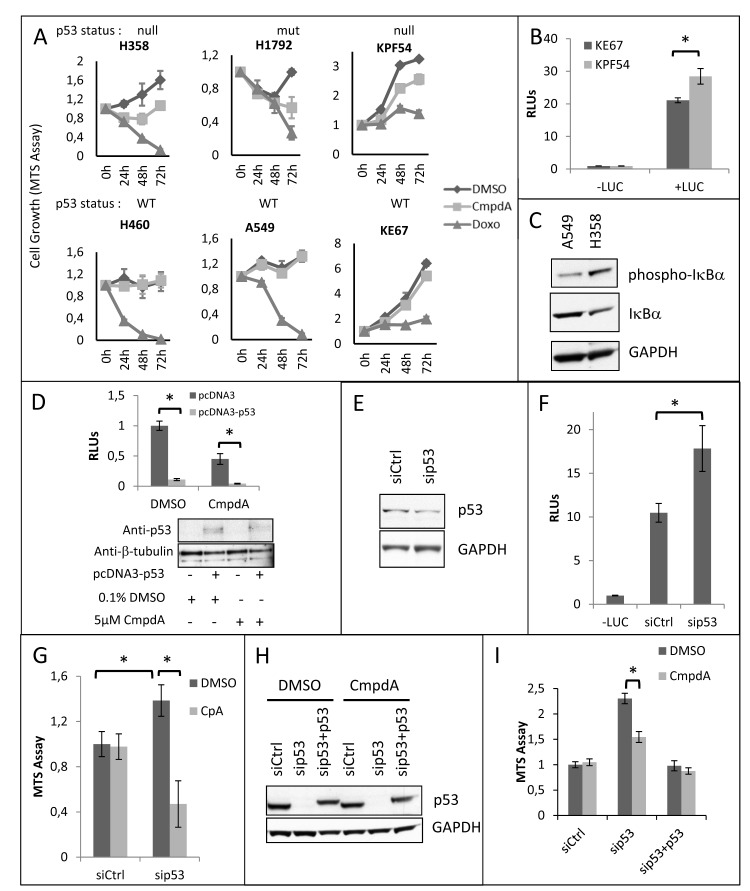
CmpdA reduced growth of KRAS positive lung cancer cells depends on loss or mutation of p53 **A)** Human and mouse KRAS positive lung cancer cell lines harboring wildtype (WT) p53 (A549, H460, KE67), mutant p53 (H1792) or lacking (null) p53 (H358, KPF54) were treated with either 0.1% DMSO or 5μM CmpdA as indicated. 2μg/mL Doxorubicin (Doxo) was used as a positive control. Cell growth was measured at the indicated timepoints using a colorimetric MTS tetrazolium assay (CellTiter 96® AQ_ueous_ One Solution Cell Proliferation Assay from Promega, Madison, WI). **B)** p53 WT KE67 and p53 null KPF54 cell lines were transfected with 100ηg of an NF-κB-responsive firefly luciferase reporter vector (3x-κB-Luc) and 5ηg of Renilla luciferase vector (pRL-TK) and NF-κB activity was analyzed by dual luciferase reporter assays. –LUC) negative control (cells transfected with pcDNA3 instead of 3x-κB-Luc and pRL-TK); RLUs) Relative luciferase units. **C)** H358 and A549 cells were analyzed for NF-κB activity by Western Blotting. Antibodies used are indicated. **D)** p53 null H358 cells were transfected with 100ηg of an NF-κB-responsive firefly luciferase reporter vector (3x-κB-Luc), 5ηg of Renilla luciferase vector (pRL-TK) and 200ηg of either empty pcDNA3 or pcDNA3-p53. After transfection, cells were treated with either 0.1% DMSO or 5μM CmpdA for 16h. NF-κB activity was analyzed by dual luciferase reporter assays. Expression of recombinant p53 was evaluated by Western Blotting. Antibodies used are indicated. RLUs) Relative luciferase units. **E)** p53 WT murine KE67 cells were transfected as described in methods with a siRNA targeting murine p53 (sip53) or non-targeting siRNA (NTctrl). At 48h these cells were transfected with 100ηg of an NF-κB-responsive firefly luciferase reporter vector (3x-κB-Luc) and 5ηg of Renilla luciferase vector (pRL-TK) and analyzed at 72h after siRNA transfection for knockdown efficiency by Western Blotting. Antibodies used are indicated. **F)** KE67 cells were transfected as described in (E) and NF-κB activity was analyzed by dual luciferase reporter assays. –LUC) negative control (cells transfected with pcDNA3 instead of 3x-κB-Luc and pRL-TK); RLUs) Relative luciferase units. **G)** KE67 cells were transfected as described in methods with a siRNA targeting murine p53 (sip53) or non-targeting siRNA (NTctrl). Subsequently cell growth was measured at 72h post-transfection using a colorimetric MTS tetrazolium assay (CellTiter 96® AQ_ueous_ One Solution Cell Proliferation Assay from Promega, Madison, WI). **H)** A549 cells were transfected as described in methods with a siRNA smartpool targeting human p53 (sip53) or a non-targeting siRNA (NTctrl). Subsequently, cells transfected with sip53 were transfected either with pcDNA3 (empty vector control) or with pcDNA3-p53 vector. Analysis of p53 knockdown and p53 re-expression was performed by Western Blotting. Antibodies used are indicated. **I)** A549 cells were transfected as described in H and cell growth was evaluated at 72h post-transfection using a colorimetric MTS tetrazolium assay (CellTiter 96® AQ_ueous_ One Solution Cell Proliferation Assay from Promega, Madison, WI). Statistical significance in all cases was measured by Student's *t*-test (*p<0.05) when compared to experimental control samples (si*Ctrl*). Error bars represent average ± 1s.d.

CmpdA sensitivity was also dependent on the status of the tumor suppressor p53. KRAS positive cell lines with wild-type (WT) p53 (A549 and H460) were less sensitive to CmpdA treatment than KRAS positive p53 null (H358) or p53 mutant (H1792) cell lines (Fig. 3A). In order to address a role for p53 in the sensitivity to IKK inhibition, we analyzed cells isolated from KRAS-induced lung cancer mouse models with WT p53 (KE67) and with deletion of the p53 gene (KPF54). Results demonstrate that the tumor cells with loss of p53 are more sensitive to IKKβ inhibition than cells with WT p53 (Fig. 3A).

Because loss of p53 can increase IKKβ activity [31] and has been shown to activate NF-κB in KRAS-transformed lung tumor cells [22], we propose that, even though NF-κB is active in all KRAS positive cell lines, loss or disruption of p53 activity would to lead to enhanced IKKβ activation and thus to enhanced CmpdA sensitivity (see discussion). In order to determine if p53 null cells have higher NF-κB activity than p53 WT cells, we performed luciferase reporter assays on murine cells derived from KRAS-induced lung tumors with either WT p53 or with loss of p53. As can be seen in Fig. 3B, KPF54 cells, which lack p53, display higher NF-κB reporter activity than KE67 cells, which express WT p53. Similar results were observed when we compared NF-κB activity between human cells with different p53 status. A549 cells, which express WT p53 have lower levels of phosphorylated IκBα (Fig. 3C), a hallmark of NF-κB activation. Finally, we expressed p53 in human H358 cells, which are p53 null, and this resulted in reduced NF-κB reporter activity (Fig. 3D).

In order to determine if the increased sensitivity to CmpdA displayed by p53 null cell lines can be caused by loss of p53, we used RNA interference to reduce p53 expression in p53 WT KE67 murine cells and in p53 WT A549 human cells. A 58% reduction in p53 expression in KE67 cells (Fig. 3E) results, not only in increased NF-κB reporter activity (Fig. 3F), but also in increased cell growth, which is reduced by CmpdA treatment (fig. 3G). Interestingly CmpdA had no effect on KE67 cells transfected with a control small interfering RNA (siRNA). Similar results were observed in A549 cells. Inhibition of p53 expression resulted in increased A549 cell growth and rendered A549 cells sensitive to CmpdA treatment (Figs. 3H and 3I). More importantly, p53 re-expression in these cells, not only reduced cell growth, but turned them insensitive to IKKβ inhibition (Figs. 3H and 3I). In order to further corroborate this data we used HCT116 colon cancer cells that harbor oncogenic KRAS and that have been engineered by homologous recombination to lose the p53 gene, making it straightforward to evaluate the effect of p53 loss in an isogenic setting. Not only is NF-κB activity higher in HCT116-p53 knockout (KO) cells than in HCT116-p53 WT cells (Supplementary Fig. S2A and S2B), but also, as assessed by MTT assays, HCT116-p53 WT are insensitive to CmpdA treatment, whereas HCT116-p53 KO cells are sensitive (Supplementary Fig. S2C). When we used clonogenic cell growth assays, both cell lines were sensitive to CmpdA treatment, but they displayed different levels of sensitivity. Whereas HCT166-p53 WT cells showed a 55% reduction in colony formation, HCT116-p53 KO cells showed an 80% reduction (Supplementary Fig. S2D). Taken together these results indicate that the status of both KRAS and the p53 tumor suppressor are important in determining sensitivity to IKKβ inhibition therapy.

**Fig 4:**
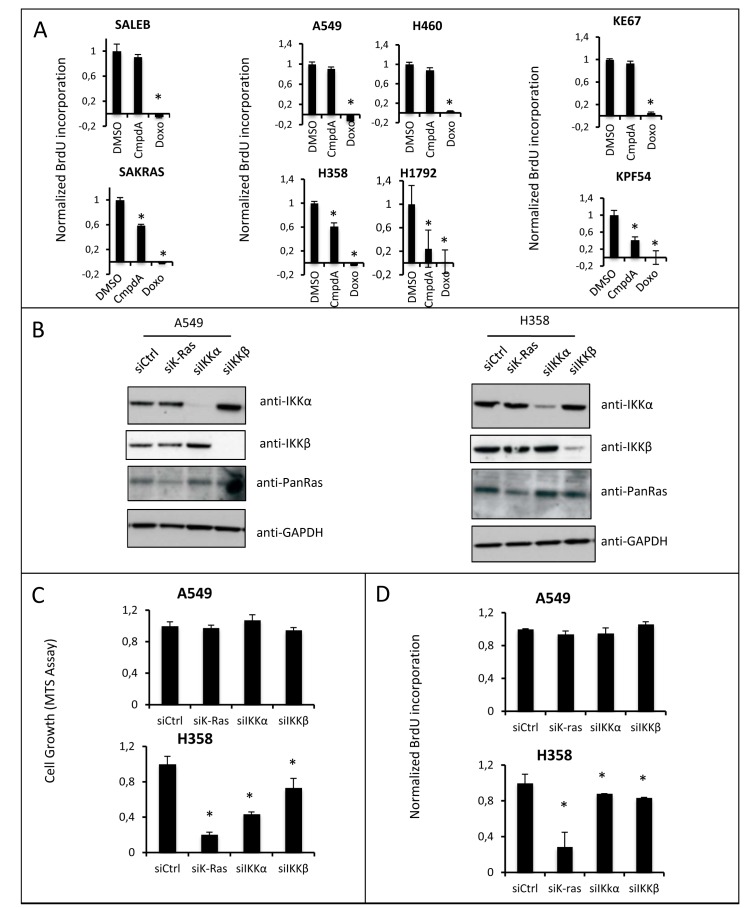
IKK targeting reduces lung cell growth by inhibiting cell proliferation **A)** Proliferation was evaluated by BrdU incorporation to measure DNA synthesis in a colorimetric assay (BrdU Cell Proliferation Assay from EMD Millipore, Billerica, MA) in all indicated cell lines after 0.1% DMSO or 5μM CmpdA treatment for 48h. 2μg/mL Doxorubicin (Doxo) was used as a positive control. **B)** The indicated cell lines were transfected as described in methods with a non-targeting control siRNA (si*Ctrl*) or with siRNA smartpools targeting KRAS (si*KRAS*), IKKβ (si*IKK*β) or IKKα (si*IKK*α) and efficacy of siRNA targeting was evaluated at 72h by Western Blotting with the indicated antibodies. **C)** Cell growth of siRNA-transfected cells (as in B) was measured using a colorimetric MTS tetrazolium assay (CellTiter 96® AQ_ueous_ One Solution Cell Proliferation Assay from Promega, Madison, WI); **D)** Proliferation of siRNA transfected cells (as in B) was evaluated at 72h by BrdU incorporation to measure DNA synthesis (BrdU Cell Proliferation Assay from EMD Millipore, Billerica, MA). Statistical significance in all cases was measured by Student's *t*-test (*p<0.05) when compared to experimental control samples (DMSO). Error bars represent average ± 1s.d.

In order to determine the mechanism leading to reduced cell growth induced by CmpdA, we analyzed both apoptosis and reduced proliferation. Interestingly, even though NF-κB is known as an antiapoptotic transcription factor, growth reduction was not caused by apoptotic cell death, as CmpdA failed to induce apoptosis in any of the cell lines (Supplementary Fig. S3A). However, reduction in cell growth in all sensitive cell lines was associated with reduced proliferation as measured by BrdU incorporation (Fig. 4A).

In order to validate IKKβ as the relevant CmpdA target in mediating reduced proliferation, we pursued siRNA experiments in H358 cells and A549 cells (Fig. 4B). The A549 cell line, which is resistant to CmpdA treatment, is also resistant to alterations in cell growth (Fig. 4C) and proliferation (Fig. 4D) upon IKKβ knockdown. On the other hand the H358 cell line, which is sensitive to CmpdA treatment, is more sensitive to loss of IKKβ by siRNA, displaying a 27% reduction in cell growth (Fig. 4C) and 20% in proliferation (Fig. 4D). The reduction in proliferation observed upon IKKβ knockdown is not as robust as that attained with CmpdA treatment (20% versus 39%). This could be caused by residual expression of IKKβ in the knockdown experiments and/or a compensatory increase in IKKα activation, which could affect cell proliferation (see below). siRNA-mediated knockdown of KRAS in H358 cells led to a greater reduction of cell growth (Figs. 4C and 4D), suggesting that KRAS activates additional pathways that contribute to cell proliferation.

Because IKKα is a member of the IKK complex and because siRNA targeting of IKKα in KRAS positive cells also reduces NF-κB activity and inhibits IκBα phosphorylation/degradation (Supplementary Fig. S1 and Fig. 1C), it is important to determine the contribution of this kinase to the proliferative phenotype of KRAS positive/p53 null lung cancer cells. Therefore, we determined if siRNA knockdown of IKKα would affect cell growth, apoptosis and proliferation. Interestingly, like inhibition of IKKβ, siRNA to IKKα in p53 WT A549 cells did not affect cell growth or proliferation, whereas in the CmpdA sensitive H358 cell line knockdown of IKKα also decreases cell growth and proliferation (Fig. 4C and 4D), suggesting that both IKKα and IKKβ contribute to promote cell proliferation. Again, consistent with the observed lack of effect of CmpdA on apoptosis, knockdown of IKKα, IKKβ or even KRAS in these cell lines, did not trigger apoptosis (Supplementary Fig. S3B).

To further corroborate these results, we examined a panel of apoptosis and proliferation-related genes in H358 cells following knockdown of KRAS, IKKα or IKKβ. Whereas, no significant changes were observed in the expression of anti-apoptotic genes *BCL2* and *CIAP2*, expression of the proliferation-related genes *E2F1* and *MYC* were affected (Supplementary Fig. S3C). Knockdown of KRAS inhibited expression of both *E2F1* and *MYC*. Interestingly, even though to a lesser extent, knockdown of either IKKα or IKKβ also inhibited *E2F1* expression. In the case of *MYC*, knockdown of IKKα or IKKβ also reduced *MYC* expression to the same extent as knockdown of KRAS. Taken together, these results indicate that both IKK forms induce NF-κB activity and proliferation downstream of RAS.

### *In vivo* administration of CmpdA reduces tumor burden in a mouse model of lung cancer triggered by KRAS activation coupled with p53 loss

**Fig 5:**
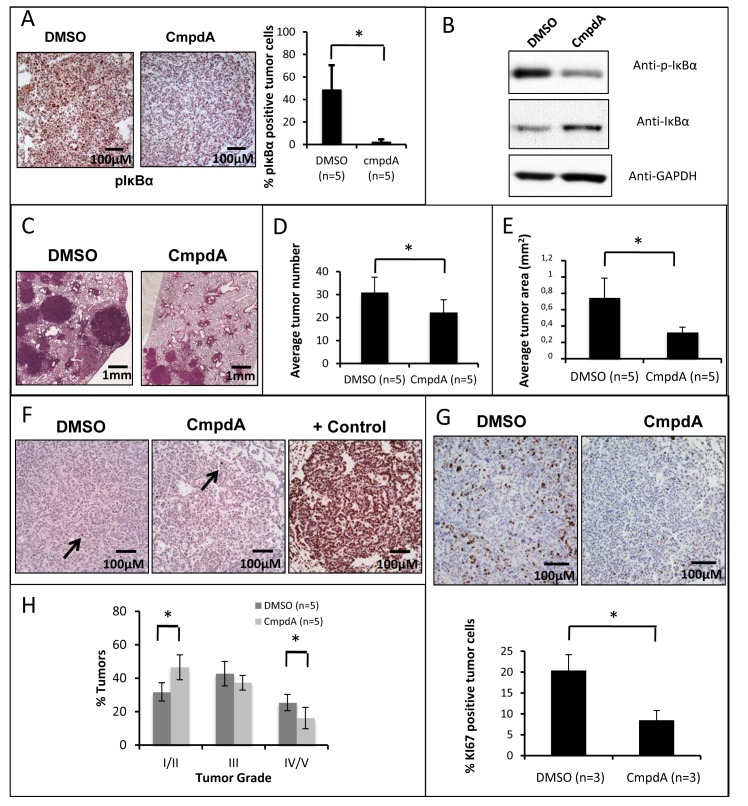
Targeting IKKβ in the KRAS^G12D^/p53Δ conditional mouse lung tumor model reduces tumor burden *in vivo* KRAS^G12D^/p53Δ mice were analyzed at 12 weeks post-infection and after 4 weeks of CmpdA or placebo (DMSO) treatment (as described in methods). **A)** Immunohistochemistry for phospho-IκBα (positive cells are brown). A representative picture of stained lung tumor slides of CmpdA-treated and placebo-treated lungs is displayed on the left panel. The graph on the right panel is a quantitative representation of the data. **B)** Western Blotting analysis of IκBα phosphorylation/degradation of CmpdA-treated and placebo-treated lung tumors. Antibodies used are indicated. **C)** A representative picture of Hematoxylin/Eosin (H/E) stained lung slides of CmpdA-treated and placebo-treated lungs. **D)** Number of KRAS-induced neoplastic lesions was determined by counting lesions in H/E stained lung sections as described in methods. **E)** Average tumor area was determined by measuring tumor area using ImageJ software. **F)** TUNEL positive cells (brown) were stained according to the Apoptag Plus *In Situ* Apoptosis Detection Kit (EMD Millipore, Billerica, MA). A representative picture of stained lung slides of CmpdA-treated and placebo-treated lungs is displayed (rare positive cells are indicated by arrows). A DNase treated slide was used as a positive control (+ control). **G)** Immunohistochemistry for KI67 (positive cells are brown). A representative picture of stained lung slides of CmpdA-treated and placebo-treated lungs is displayed on the top panel. The graph on the bottom panel is a quantitative representation of the data. **H)** Analysis of lung tumor grade was performed on H/E stained lung sections as described in methods. Statistical significance in all cases was measured by Student's *t*-test (*p<0.05) when compared to experimental control samples (DMSO). Error bars represent average ± 1s.d.

Given that CmpdA could decrease cell growth and proliferation of different cell lines with oncogenic *KRAS* mutations and loss or disruption of p53, we decided to test in a preclinical mouse model if this inhibitor would have similar effects *in vivo*. For that purpose, we used oncogenic KRAS inducible Lox-Stop-Lox (*LSL*) *Kras*^G12D^ mice with conditional inactivation of p53 [32]. In this model, both expression of oncogenic KRAS^G12D^ and inactivation of p53 are triggered by Cre-mediated recombination. Lung tumors were induced in this model by intranasal administration of Cre-expressing adenovirus (AdCre) as previously described [32]. Infected mice will be referred to as KRAS^G12D^/p53Δ mice. We administered CmpdA or placebo beginning at 8 weeks post-infection (when lung tumors are already present) for 4 weeks daily. CmpdA was well tolerated both in the tumor bearing animals as well as in healthy controls. At the endpoint, the animals were euthanized, and the tumors of CmpdA-treated animals were compared to the tumors of placebo-treated animals. To assess IKKβ inhibition in the tumors, we performed phospho-IκBα immunohistochemistry. Consistent with an inhibitory effect on IKK, tumors of CmpdA-treated animals have a dramatically reduced percentage of phospho-IκBα positive tumor cells (Fig. 5A). Decreased IκBα phosphorylation was confirmed by western blotting, as well as increased levels of total IκBα (Fig. 5B), consistent with reduced IKK activity. Even though tumors were still present at the end of treatment, CmpdA-treated animals exhibited significantly decreased tumor burden (Fig. 5C-E). We observed, not only a small (but significant) decrease in tumor number (Fig. 5D), but also a significant decrease in average tumor area (Fig. 5C and E). Taken together these results indicate that CmpdA can reduce KRAS-induced lung tumor growth in a p53 null context.

In order to determine if this reduction in tumor growth was due to tumor cell apoptosis or reduced tumor cell proliferation, we performed TUNEL staining of lung slides, as well as KI67 staining. Consistent with our cell-based experiments, we saw no change in apoptotic cells between CmpdA and DMSO treated animals (Fig. 5F). In fact, most tumors were TUNEL negative. On the other hand KI67 staining was markedly reduced in CmpdA-treated animals (Fig. 5G), indicating that CmpdA reduces lung tumor proliferation. In addition to slowing tumor growth, CmpdA also slows tumor progression. A histopathological analysis revealed that CmpdA treated mice display a higher percentage of grade I/II tumors and lower percentages of grade IV/V tumors (Fig. 5H).

Interestingly, the reduction in proliferative index and tumor area in mouse lung tumors treated with CmpdA was much more pronounced than the loss of growth and proliferation inhibition seen in our cell-based experiments, which led us to hypothesize that the effect of CmpdA on tumor burden might not be exclusively tumor-intrinsic. In fact, NF-κB is an important transcription factor driving inflammation and CmpdA has shown anti-inflammatory properties in a previous study [33]. Because inflammatory cells are important components of the tumor microenvironment, which can cooperate with cancer cells to promote oncogenesis [34], we investigated whether CmpdA would affect the number of inflammatory cells in the lungs of mice treated with CmpdA. Indeed, CmpdA treatment reduces the number of F4/80-positive macrophages in tumor-bearing lungs (Supplementary Fig. S4A).

Previous studies targeting IKKβ in tumor models did not address the role of IKKβ inhibition on tumor angiogenesis. Because RAS can promote tumor angiogenesis through the induction of the IL-8 cytokine [35], and because IL-8 is upregulated by KRAS in an IKKβ-dependent manner [23], we hypothesized that CmpdA would inhibit tumor angiogenesis. We, therefore, used immunohistochemistry for a specific endothelial marker (CD31) to compare tumor vessel density in CmpdA-treated versus placebo-treated animals. As can be seen in Supplementary Fig. S4B, CmpdA treatment reduces tumor vessel density. These results indicate that this inhibitor affects not only tumor cells, but also the tumor microenvironment, which is an important variable to be considered for therapy design.

Taken together these results suggest that CmpdA or other highly specific IKKβ or IKKα inhibitors might produce a clinical benefit for patients with KRAS-induced lung cancer with disrupted p53 function.

## DISCUSSION

KRAS-induced cancers are very common, yet despite intense efforts, effective therapies for these malignancies are sofar unavailable. Approaches to target KRAS directly have sofar been unsuccessful [7] and indirect targeting of KRAS through its downstream effectors has proven to be challenging, as KRAS regulates many pathways that impinge on the oncogenic phenotype. Effective KRAS targeting will likely involve combined inhibition of these pathways. Therefore, a better understanding of the oncogenic pathways triggered by KRAS is warranted, as it will identify additional druggable targets that will increase the possibilities for combined therapy design.

To more fully understand the oncogenic pathways triggered by KRAS, we and others have shown that oncogenic KRAS activates NF-κB in lung tumors, which in turn potentiates lung tumorigenesis. We have shown that KRAS activates an NF-κB EGFP reporter in lung tumors *in situ* [23]. In addition, loss of the main NF-κB subunit p65 in KRAS-lung tumors was associated with a reduction in tumor number, spread and grade [23]. Meylan *et al* [22] have shown the expression of a degradation-resistant form of the NF-κB inhibitor IκBα, also led to reduced KRAS-induced lung tumor growth. Barbie *et al* [24] have shown in a variety of different cell line models, that loss of c-REL, another member of the NF-κB family, was synthetically lethal with oncogenic KRAS. Finally, we had originally shown that the combined loss of c-REL and p65 reduced RAS-induced colony formation more than loss of p65 alone [36]. These findings indicate that activation of NF-κB by KRAS is an important event in tumorigenesis, and that targeting this pathway might prove beneficial in therapy.

Interestingly, different molecular pathways with druggable targets have been reported linking oncogenic Ras to NF-κB activation. Chien *et al* [37] demonstrated that activation of TBK1 by RalB, a Ras effector, is required for Ras-induced transformation. Barbie *et al* [24] have shown that TBK1 loss leads to synthetic lethality of a variety of KRAS transformed cells. Recently, our lab has shown that in pancreatic cancer KRAS utilizes GSK3α to promote both canonical and non-canonical NF-κB activation [38]. Duran *et al* [25] demonstrated that activation of NF-κB by KRAS involves p62-mediated TRAF6 ubiquitination and activation of IKK. Interestingly, TRAF6 has been shown to be amplified in lung cancers and to function as an oncogene required for Ras-induced transformation [39]. Loss of p62, not only reduces tumor formation in a KRAS-induced lung cancer mouse model [25], but has also been implicated in sustaining NF-κB activity induced by KRAS-IL1α in pancreatic cancer [27].

Given the above mentioned data linking KRAS to activation of the canonical IKK complex in lung cancer, and given that we had previously shown that increased NF-κB activity in KRAS-driven mouse lung tumors were associated with increased phosphorylation of IκBα, and that KRAS-induced NF-κB activity in lung cells required IKKβ [23], the main catalytic subunit of the canonical IKK complex, we hypothesized that a reasonable druggable target in the KRAS-induced NF-κB activation pathway in lung cancer would be the IKKβ kinase. Recently, further corroborating our hypothesis, Xia *et al* [28] have demonstrated that genetic deletion of IKKβ reduces lung tumorigenesis in a mouse model. Another important consideration is that, even though NF-κB is ubiquitously expressed, under normal conditions it is usually present in an inactive form. NF-κB activation is important mainly under stress conditions such as during the inflammatory and innate immune responses. Therefore, systemic NF-κB inhibition therapy (by blocking IKKβ activity) has the potential to have tolerable side effects and thus provide a broader therapeutic window.

In order to test IKKβ inhibition therapy as an approach to treat KRAS-induced lung cancer we used CmpdA, a highly specific IKKβ inhibitor [29]. Using this inhibitor, we had previously shown that it blocks NF-κB activity in KRAS-transformed primary lung cells (SAKRAS) cells and H358 cells [23]. Here, we demonstrated that CmpdA inhibits NF-κB activity in other KRAS positive lung cancer cell lines as well (Fig. 1A). While CmpdA is a highly specific IKKβ inhibitor, it has been published that CmpdA has an approximately 70-fold lower activity on IKKα [29]. Even though we believe that the major effect of CmpdA is on IKKβ, we cannot rule out that it may also suppress the activity of IKKα.

Interestingly, NF-κB activity was inhibited in these cells by siRNA to either IKKβ or IKKα (Supplementary Fig. S1). While the result with knockdown of IKKβ was not surprising, the result with knockdown of IKKα was surprising since it is widely considered that IKKβ is dominant in the canonical NF-κB pathway [30]. Nonetheless, we had previously found that IKKα is involved in canonical NF-κB activation downstream of Her2/ErbB2 [40]. Additionally, knockdown of IKKα in H358 cells does not affect p100 processing (not shown), but it decreases IκBα phosphorylation (Fig. 1C), indicating that in these cells IKKα is largely affecting canonical NF-κB signaling.

Interestingly, CmpdA treatment reduced cell growth leading to a cytostatic effect. This effect was not only dependent on KRAS status as expected (Fig. 2), but also dependent on loss or altered function of the tumor suppressor p53 (Fig. 3). We have shown that inhibition of p53 expression in p53 WT lung cancer cells promotes sensitivity to CmpdA treatment and that p53 re-expression reverses this effect (Fig.3). In addition p53 knockout HCT116 colon cancer cells, which harbor oncogenic KRAS, are also more sensitive to CmpdA treatment than p53 WT HCT116 cells (Supplementary Fig. S2). This p53 dependency was unexpected, as we have shown that targeting p65/RELA in a p53 competent KRAS-induced lung cancer mouse model reduces tumorigenesis [23] and loss of IKKβ in a p53 competent RAS-induced melanoma model [26] or in a p53 competent KRAS-induced pancreatic cancer model [27] also reduces tumorigenesis. One explanation for this dependency on p53 status is that, even though KRAS activates NF-κB, loss of p53 has been shown to further enhance NF-κB activation by post-translational activation of IKKβ [31]. In fact, Meylan *et al* [22] showed robust NF-κB activation in KRAS positive cells upon loss of p53. It is possible that the KRAS-induced NF-κB activity level in these p53 WT lung cells is not sufficient to render them intrinsically sensitive to CmpdA treatment in these *in vitro* studies. In fact, we have shown that inhibition of p53 expression in human and murine lung cancer cells leads to enhanced NF-κB activity and likewise, that expression of p53 in p53 null H358 cells reduces NF-κB activity (Fig.3). We note that HCT116 p53^WT^ cells do exhibit a level of sensitivity to CmpdA, which may be due to the fact that these cells have low p16 expression, which has been published to suppress NF-κB [41]. It is not possible to predict how p53 WT KRAS-induced lung tumors would respond to IKKβ targeting *in situ*, as all *in vivo* experiments targeting this kinase in lung cancer were performed in models with disrupted p53 function [28,42]. We propose that combined activation of KRAS and loss of p53 in lung tumor cells would lead to enhanced IKK activity and to increased CmpdA sensitivity *in vivo*.

In order to investigate if IKKβ inhibition therapy would be effective *in vivo*, we treated KRAS^G12D^/p53Δ mice with CmpdA. CmpdA not only inhibited IKK activity in lung tumors, but also significantly reduced tumor burden, tumor proliferation and slowed tumor progression (Fig. 5). In accordance with our data, Xue *et al* [42] demonstrated that treatment of KRAS^G12D^/p53Δ mice treated with bortezomib, a proteassome inhibitor that prevents degradation of IκBα, also reduces tumor growth. Although proteasome inhibition leads to complex cellular phenotypes, IKK inhibitors were also effective in prolonging survival of KRAS induced lung tumor models [28,42]. Finally, genetic targeting of IKKβ in melanoma, pancreatic and lung cancer mouse models also affected the kinetic of tumor development [26-28]. These results, together with ours, suggest that therapeutic strategies aimed at blocking NF-κB activity should prove beneficial for KRAS-induced lung cancer therapy, especially in patients with simultaneous loss or mutation of p53.

Interestingly, whereas loss of NF-κB p65/RELA in KRAS-induced lung tumors is associated with an apoptotic response [23], inhibition or loss of IKKβ does not induce apoptosis in cell lines or in KRAS induced lung tumors (Supplementary Fig. S3 and Fig. 4, [28]). This puzzling result can be explained by recent findings demonstrating that kinase inhibition can lead to activation of compensatory pathways that can affect the biological readout [43-46]. In this regard, targeting NF-κB directly through p65/RELA loss [23] or expression of a degradation resistant form of IκBα [22] may lead to an apoptotic response, whereas targeting the upstream IKKβ kinase could activate compensatory pathways that would eliminate this response, and could even explain the acquisition of therapy resistance, which was observed upon treatment with the IKK inhibitor Bay-117082 [42]. Nonetheless, the antiproliferative effect of IKKβ targeting is retained.

In addition to promoting intrinsic tumor cell proliferation, RAS oncoproteins can influence the tumor microenvironment by inducing secretion of pro-inflammatory and pro-angiogenic cytokine IL-8 [35,47]. It is well known that *IL-8* is a NF-κB target gene [48,49] and we have shown that in KRAS-transformed lung cells, *IL-8* expression depends on IKKβ [23]. Here we show that IKKβ inhibition by CmpdA treatment affected the microenvironment of KRAS-induced lung tumors, reducing tumor-related inflammation and angiogenesis (Supplementary Fig. S4). It remains to be determined if CmpdA causes a reduction in the ability of tumor cells to recruit inflammatory and endothelial cells to the tumors or if it directly affects activation and migration of these cells. It is likely that both possibilities coexist. In the case of inflammation, CmpdA has been shown to have anti-inflammatory effects [33], and genetic loss of IKKβ in mouse lung tumors is associated with a reduced inflammatory infiltrate [28]. In the case of angiogenesis, expression of a dominant negative form of IKKβ in endothelial cells inhibits tube formation [50]. Even though previous studies with genetic targeting of IKKβ in tumor mouse models did not address the effect of IKKβ deletion on tumor angiogenesis, it has been demostrated that IKKβ can promote tumor angiogenesis by phosphorylating TSC1 and activating mTOR in tumor cells [51]. Finally, we cannot disregard the possibility that the reduced vessel density observed results from reduced tumor-associated inflammation, as tumor associated macrophages can further stimulate secretion of pro-angiogenic cytokines by tumor cells [52].

Interestingly, even though treatment of mice with the IKKβ inhibitor TPCA-1 was associated with toxicity [28], this study shows that CmpdA is well tolerated in mice. In fact, CmpdA is a highly specific IKKβ inhibitor, which can inhibit IKKα at a 70-fold higher concentration, but does not affect activity of other related serine threonine kinases including TBK1 and IKKε [29]. Other studies using this inhibitor also report that CmpdA is not associated with significant toxicity in mice [29,33,53], indicating CmpdA as a promising IKKβ inhibitor to be used in clinical trials for cancer therapy.

It is important to acknowledge that CmpdA treatment does not lead to complete tumor regression. Alternative pharmacological and genetic approaches to target the NF-κB pathway in KRAS-driven lung cancer also do not completely eliminate tumorigenesis [23,28]. Interestingly, we have shown that loss of KRAS led to greater reduction of cell growth and proliferation than loss of either IKKβ or IKKα, suggesting that KRAS activates additional and/or alternative pathways that contribute to cell proliferation. Effective KRAS targeting will likely involve combined inhibition of these pathways.

In conclusion, we have identified IKKα and IKKβ as promising druggable targets to inhibit NF-κB activity downstream of KRAS and loss of p53. We have also demonstrated in a preclinical model of KRAS-induced lung cancer with loss of p53 that IKKβ inhibition therapy is safe in mice and, even though it is not curative, it shows efficacy in slowing tumor growth. Taken together, our results indicate that IKKα or IKKβ inhibition therapy might prove to be a promising approach to be validated in clinical trials, particularly using combination strategies to treat KRAS-induced lung cancer with loss of the tumor suppressor p53.

## MATERIALS AND METHODS

### Cell culture

Cell passages were kept to a minimum and no cells were passaged continuously for more than six months. Low passage SALEB and SAKRAS cells were cultured in serum-free bronchial epithelium growth medium (BEGM, Clonetics-Lonza, Allendale, NJ). These cells were originally selected in medium containing a triple antibiotic cocktail and subsequently characterized by real-time PCR for expression of the genes used for immortalization and transformation [54]. Short Tandem Repeat-DNA profile authenticated NCI-H358, A549, NCI-H460 and NCI-H1792 cells were obtained from ATCC (Manassas, VA) and maintained in RPMI 1640 (Invitrogen, Carlsbad, CA) supplemented with 10% FBS (Sigma-Aldrich, St. Louis, MO). KE67 cells were generated and characterized in the laboratory [23]. KPF54 cells were generated as described for KE67 cells from lung tumors of oncogenic KRAS inducible Lox-Stop-Lox (*LSL*) *Kras*^G12D^ mice with conditional inactivation of p53 [32]. Their origin was authenticated by PCR of the excised *Kras* and *p53* alleles. They were maintained in Dulbecco's Modified Eagle's Medium (DMEM, Invitrogen, Carlsbad, CA) supplemented with 10% FBS and 0.5mM 2-mercaptoethanol (both from Sigma-Aldrich, St. Louis, MO).

### Transfections and Reporter Assays

siRNA transfections were performed as previously described [55] with 100ηM of either a non-targeting siRNA control or siRNA smart pools targeting p53, IKKβ, IKKα or KRAS (Dharmacon/Thermo Scientific, Pittsburgh, PA). For p53 knockdown studies in murine cells, we used 50ηM of p53 siRNA or non-targeting control siRNA (Ambion/Life Technologies, Grand Island, NY). Plasmid DNA transfection using a pcDNA-p53 expression vector (Addgene, Cambridge, MA) were performed with Lipofectamine LTX (Life technologies, Grand Island, NY) according to the manufacturer´s instructions. Combined siRNA/plasmid transfections were performed sequentially, with the plasmid transfection performed 48h after the siRNA transfection. Dual Luciferase Reporter assays were performed as described [55]. For pharmacological studies, cells were treated with CmpdA or vehicle control as indicated (see figure legends). Relative light units were measured on an Lmax Microplate Luminometer (Molecular Devices, Sunnyvale, CA).

### 3-(4,5-dimethylthiazol-2-yl)-5-(3-carboxymethoxyphenyl)-2-(4-sulfophenyl)-2H-tetrazolium salt (MTS) assay

The MTS assay was performed using the Cell Titer 96 AQueous One Solution Cell Proliferation assay (Promega, Madison, WI) according to the manufacturer's protocol. The reduction of MTS to formazan was measured colorimetrically at 490nm on a VersaMax Microplate Reader (Molecular Devices, Sunnyvale, CA).

### BrdU Incorporation Assay

BrdU incorporation was evaluated using the BrdU Cell Proliferation Assay (EMD Millipore, Billerica, MA) following the manufacturer's instructions. Cells were incubated with BrdU for 2h prior to harvesting for analysis. BrdU incorporation was measured colorimetrically on a VersaMax Microplate Reader (Molecular Devices, Sunnyvale, CA) at 450nm.

### Western Blotting

Western Blotting was performed as described [55]. The antibodies used and respective catalog numbers were as follows: anti-IKKβ (05-535), anti-IKKα (05-536) and anti-pan-RAS (OP-40) were from EMD Millipore (Billerica, MA); anti-IκBα (4812) and anti-phospho(s32/36)IκBα (9246) were from Cell Signaling (Danvers, MA); we also used anti-GAPDH (sc-25778) and anti-p53 antibodies (cs-2527, Cell Signaling, Danvers, MA and sc-98, Santa Cruz Biotechnology, Santa Cruz, CA).

### Animal husbandry and Cre-expressing adenovirus (adenocre) administration

Lox-stop-lox (*LSL*) *Kras*^G12D^/*p53*^fl/fl^ mice, generated by crossing strains B6.129S4-*Kras^tm4Tyj^* and B6.129P2-*Trp53^tm1Brn31^* were housed in pathogen-free conditions according to the protocols approved by the UNC Institutional Animal Care and Use Committee. Lung tumor induction was performed by intranasal administration of 1x10^7^ plaque forming units (pfus) of adenocre (Gene Transfer Vector Core, University of Iowa, Iowa City, IA) in selected animals at 8 weeks of age, as described [56].

### *In vivo* Administration of CmpdA:

10mg/kg of IKKβ inhibitor CmpdA (Bayer [29], Pittsburgh, PA) was administered intraperitoneally daily for 4 weeks, starting at 8 weeks post-infection. Dimethyl sulfoxide (DMSO, Sima-Aldrich, St. Louis, MO) was used as vehicle control. To minimize the risk of DMSO-induced toxicity, we used CmpdA at a concentration of 5mg/mL to reduce the volume used to perform injections. All mice were euthanized at the end of treatment (12 weeks post-infection).

### Histopathological analysis

Mice were euthanized by intraperitoneal administration of 250mg/kg of freshly prepared avertin followed by surgical resection of the portal vein. Lungs were perfused with saline and inflation-fixed overnight with formalin 10%. Fixed tissues were be embedded in paraffin, sectioned at 5 micrometer thickness and stained with hematoxylin/eosin.

### Terminal deoxynucleotidyl transferase–mediated dUTP nick end labeling (TUNEL) assay

Tissue sections were deparaffinized, rehydrated, and stained according to the ApopTag Plus *In Situ* Apoptosis Detection kit (EMD Millipore, Billerica, MA) instructions.

### Immunohistochemistry:

Formalin-fixed, paraffin-embedded tissue sections were stained with rat monoclonal anti-KI67 antibody (Clone TEC3, DakoCytomation, Carpinteria, CA) diluted 1:25, or mouse monoclonal anti-phospho-IκBα-Ser32/36 (9246, Cell Signaling, Danvers, MA) diluted 1:100 using the Rat Vector Elite ABC Kit (Vector Laboratories, Burlingame, CA) or the Mouse on Mouse (M.O.M) Peroxidase Kit (Vector Laboratories, Burlingame, CA), following the manufacturer´s protocol. Quantitation of staining results was perfomed using ImageJ software to count positive cells.

### Tumor number and grade analysis

Slides from each lung lobe (same orientation and level section used for each lobe) were scored for tumor number, area and grade blindly. For tumor number and area, low magnification pictures were taken in a Olympus BX61 upright fluorescence microscope (Olympus, Center Valley, PA) and images were analyzed using ImajeJ software. For analysis of tumor grade, slides were scanned with a 40X magnification resolution using a Scanscope CS Digital Scanner (Aperio, Vista, CA). Tumors were scored using ImageScope software (Aperio, Vista, CA). Each tumor was given a score of 1 to 5 based on previously described criteria [32].

### Statistics

All values are presented either as mean ± 1SD or as representative images of at least three independent experiments. In the case of animal studies, at least three mice of each different genotype were analyzed. All comparisons were made using the unpaired Student *t* test for samples with unequal variance. Differences were considered statistically significant at *p* ≤ 0.05 (indicated by asterisks).

## SUPPLEMENTARY FIGURES


