# Supplementary material for: IKK is a therapeutic target in KRAS-Induced lung cancer with disrupted p53 activity
Source: Genes Cancer. 2014 Jan;5(1-2):41–55. doi: 10.18632/genesandcancer.5 (PMC4063255; doi:10.18632/genesandcancer.5)
Supplement: Supplementary file 1 [file ganc-05-041-s001.pdf]

IKK is a therapeutic target in KRAS-induced lung cancer with disrupted p53 activity

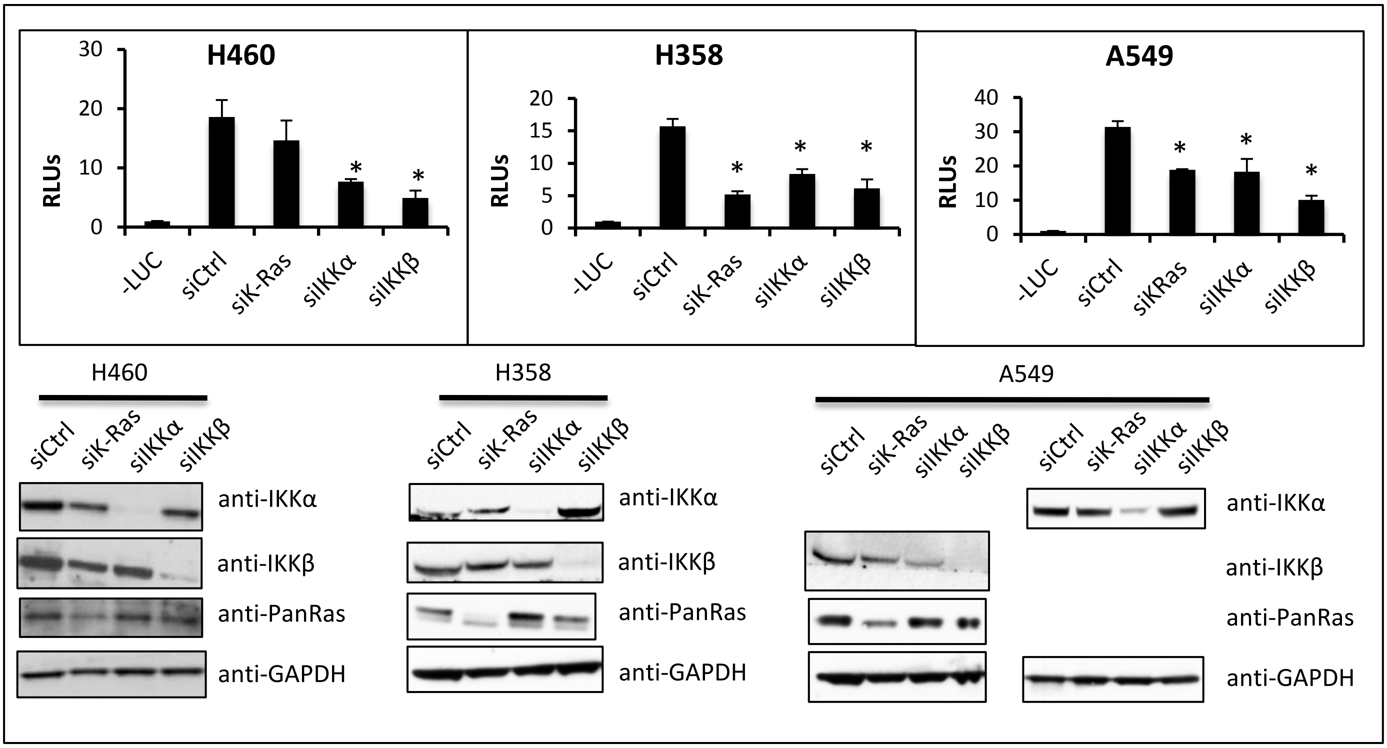

Supplemental Figure 1: Targeting IKKβ and IKKα by RNA interference reduces NF-κB activity in KRAS positive lung cells. The indicated cells were initially transfected with 100nM siRNA smartpools targeting KRAS (si*KRAS*), IKKα (si*IKK*α), IKKβ (si*IKK*β), or nontargeting control (si*Ctrl*). At 48h posttransfection, the cells were transfected with 100[illegible]g of an NF-κB-responsive firefly luciferase reporter vector (3x-κB-Luc) and 5[illegible]g of Renilla luciferase vector (pRLTK) and analyzed at 72h after the initial transfection for NF-κB luciferase reporter activity (upper panel) and for efficacy of siRNA targeting by Western Blotting (bottom panel). –LUC) negative control (cells transfected with pcDNA3 instead of 3x-κB-Luc and pRL-TK); RLUs) Relative luciferase units. Images shown are representative of three independent experiments and statistical significance was measured when appropriate by Student's *t*-test (*p<0.05) when compared to experimental control samples (DMSO or si*Ctrl*). Error bars represent average ± 1s.d.

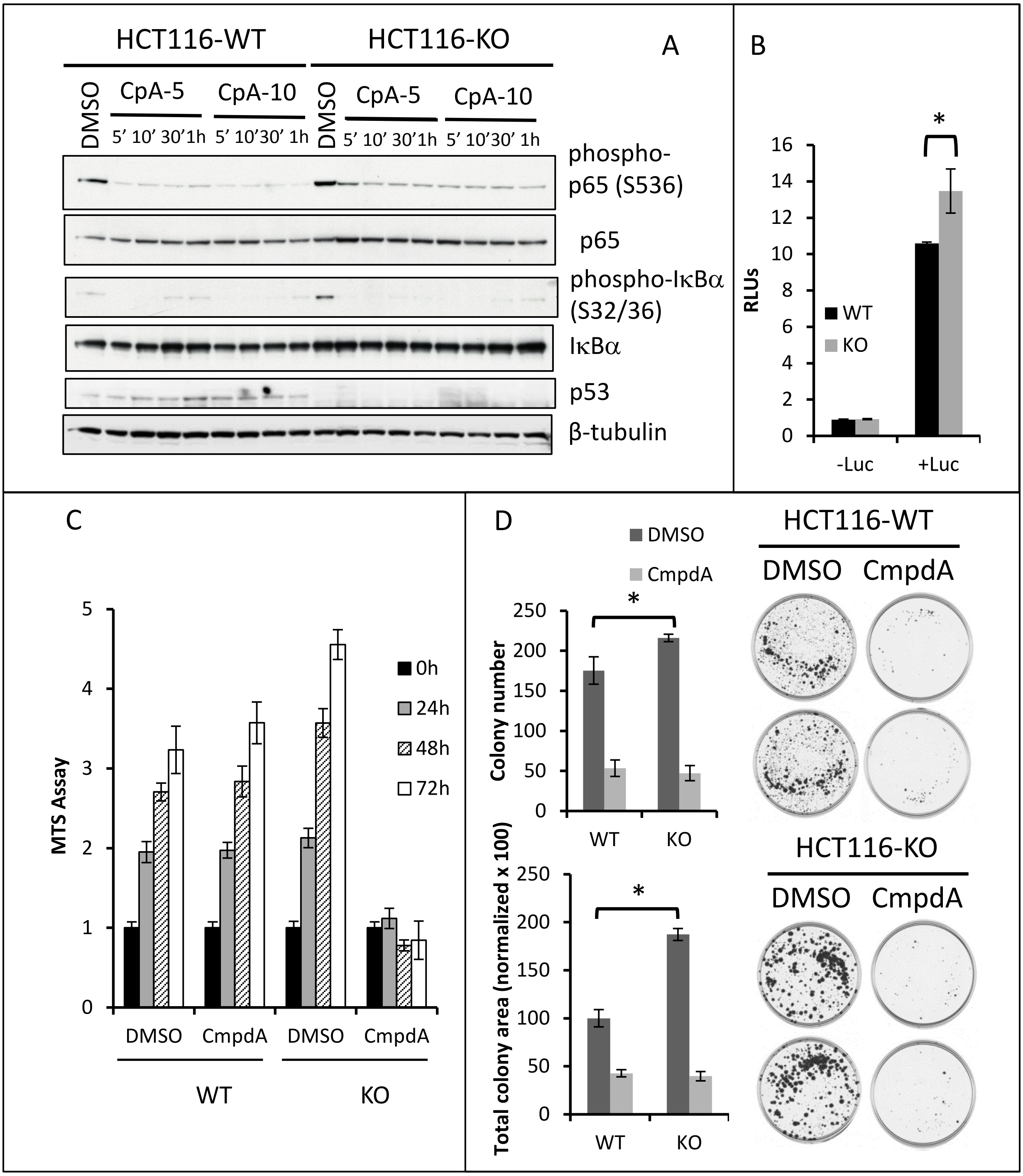

A
HCT116-WT
HCT116-KO
DMSO
CpA-5
CpA-10
5' 10' 30' 1h
phospho-p65 (S536)
p65
phospho-IκBα (S32/36)
IκBα
p53
β-tubulin
B
RLUs
WT
KO
-Luc
+Luc
*
C
MTS Assay
0h
24h
48h
72h
DMSO
CmpdA
WT
KO
D
DMSO
CmpdA
Colony number
Total colony area (normalized x 100)
WT
KO
*
HCT116-WT
HCT116-KO

Supplemental Figure 2: CmpdA reduces growth of KRAS positive HCT116 colon cancer cells with knockout of p53. A) HCT116-p53WT and HCT116-p53KO cells were treated with 0.1% DMSO, 5µM CmpdA (CpA-5) or 10µM CmpdA (CpA- 10) for the indicated times. NF-κB activity was analyzed by Western Blotting. Antibodies used are indicated. **B)** HCT116-p53WT and HCT116-p53KO cells were transfected with 100ng of an NF-κB-responsive firefly luciferase reporter vector (3x-κB-Luc) and 5ng of Renilla luciferase vector (pRL-TK) and dual luciferase reporter activity was analyzed after 16h. -LUC) negative control (cells transfected with pcDNA3 instead of 3x-κB-Luc and pRL-TK); RLUs) Relative luciferase units. **C)** HCT116-p53WT and HCT116-p53KO cells were treated with either 0.1% DMSO or 5µM CmpdA as indicated. Cell growth was measured at the indicated timepoints using a colorimetric MTS tetrazolium assay (CellTiter 96® Aqueous One Solution Cell Proliferation Assay from Promega, Madison, WI). **D)** HCT116- p53WT and HCT116-p53KO cells were plated for clonogenic assays as described in supplemental methods and treated with either 0.1% DMSO or 5µM CmpdA for 14 days. Colonies formed were stained with crystal violet, counted and the total colony area was measured. Images shown are representative of three independent experiments and statistical significance was measured when appropriate by Student's *t*-test (*$p<0.05$) when compared to experimental control samples (DMSO or si*Ctrl*). Error bars represent average ± 1s.d.

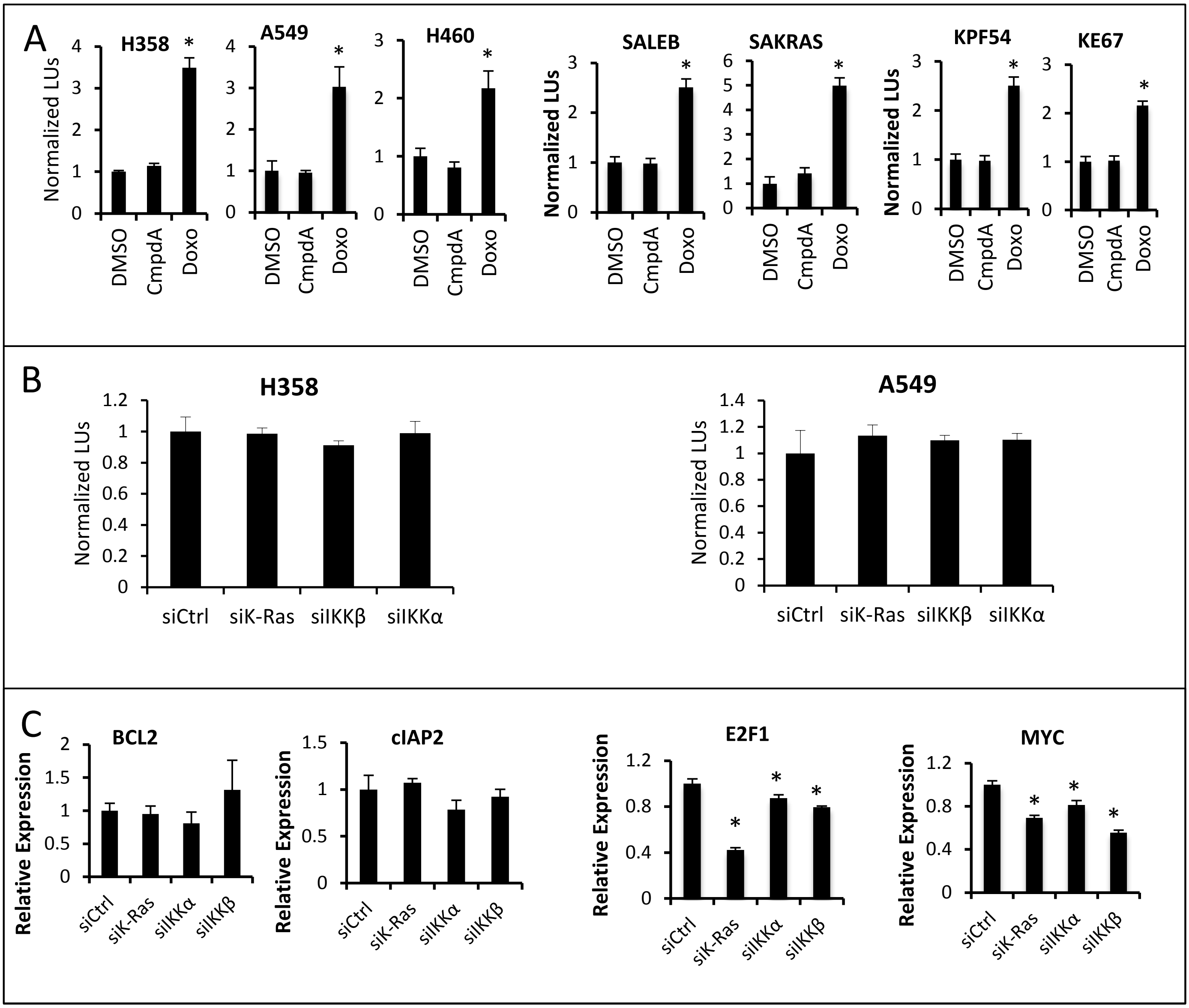

Supplemental Figure 3: Targeting IKKβ or IKKα does not trigger apoptosis of lung cancer cells. A) The indicated cell lines were treated with either 0.1% DMSO or 5µM CmpdA for 48h. Apoptosis was evaluated by measuring Caspase 3/7 activity in a luminescence-based assay (Caspase 3/7 Glo Assay, Promega, Madison, WI). Treatment with 2µg/mL Doxorubicin (Doxo) for 48h was used as a positive control. LUs) Light units. **B)** The indicated cell lines were transfected with the indicated siRNAs and apoptosis was evaluated 72h after transfection by measuring Caspase 3/7 activity in a luminescence-based assay (Caspase 3/7 Glo Assay, Promega, Madison, WI). LUs) Light units. **C)** Expression of *BCL2*, *CIAP2, E2F1* and *MYC* was analyzed by real-time quantitative PCR in H358 cells 48h after siRNA transfection. Statistical significance in all cases was measured by Student's *t*-test (*$p<0.05$) when compared to experimental control samples (DMSO). Error bars represent average ± 1s.d.

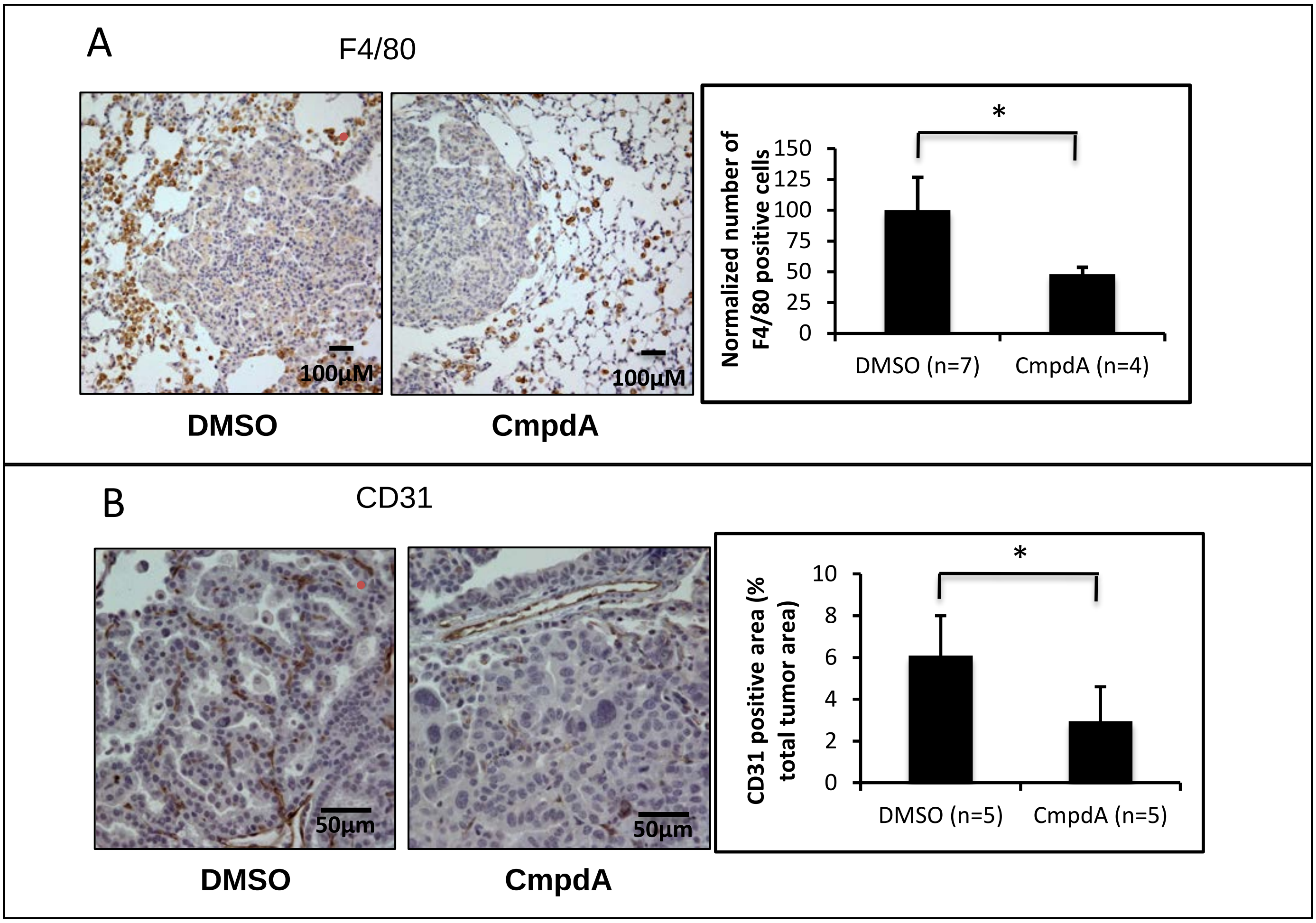

Supplemental Figure 4: Targeting IKKβ in the KRASG12D/p53Δ conditional mouse model reduces lung tumor inflammation and angiogenesis *in vivo*. Immunohistochemistry for F4/80 **(A)** and CD31 **(B)** (positive cells are brown). A representative picture of stained lung slides of CmpdA-treated and placebotreated lungs is displayed on the left panels. The graphs on the right panels are quantitative representations of the data. Statistical significance in all cases was measured by Student's *t*-test (*p<0.05) when compared to experimental control samples (DMSO). Error bars represent average ± 1s.d.

SUPPLEMENTAL MATERIALS AND METHODS

Cell culture. Cell passages were kept to a minimum and cells were not passaged continuously for more than six months. HCT116-p53 WT and HCT116-p53 knockout cells were a kind gift from Dr. Yanping Zhang. They were maintained in Dulbecco's Modified Eagle's Medium (DMEM, Invitrogen, Carlsbad, CA) supplemented with 10% FBS (Sigma-Aldrich, St. Louis, MO).

Clonogenic Assay. 5x102 HCT116-p53WT and HCT116-p53KO cells were plated in 60mm dishes and cultured for 14 days. 24h after plating, the cells were treated with either 0.1% DMSO (vehicle control) or 5µM CmpdA. The supplemented media was replaced every other day. After 14 days, the cells were fixed in ice-cold methanol for 10 minutes and subsequently stained with 0.5% crystal violet for 10 minutes. Quantitation of staining results was perfomed using ImageJ software.

Caspase 3/7 Glo Apoptosis Assay. Apoptosis was evaluated using the Caspase 3/7 Glo Assay System (Promega, Madison, WI) following the manufacturer's instructions. Luminescent caspase activity was detected on an Lmax Microplate Luminometer (Molecular Devices, Sunnyvale, CA).

RNA isolation and Real-time PCR analysis. Total RNA was prepared using TRIZOL reagent (Invitrogen, Carlsbad, CA) following the manufacturer's protocol and real-time PCR analysis was performed in an ABI 7000 Sequence Detection System using TaqMan Gene Expression Assay primer-probe sets (all from Applied Biosystems/Life technologies, Grand Island, NY) for *BCL2* (Hs00608023_m1), *CIAP2* (Hs00154109_m1), *E2F1* (Hs00153451_m1) and *MYC* (Hs00153408_m1). Relative quantitation was determined by the ΔΔ*Ct* method using *GUSB* (Hs99999908_m1) as the endogenous control.

Immunohistochemistry: Formalin-fixed, paraffin-embedded tissue sections were stained with rat monoclonal anti-F4/80 antibody (ab6640, Abcam, Cambridge , MA) diluted 1:100 or with rabbit polyclonal anti-CD31 antibody (ab28364, Abcam, Cambridge, MA) using the Rat or Rabbit Vector Elite ABC Kit (Vector Laboratories, Burlingame, CA), following the manufacturer´s protocol. Quantitation of staining results was perfomed using ImageJ software to count positive cells (F4/80) or measure positive area (CD31).
